# Detection of Genetic Mutations by Next-Generation Sequencing for Predicting Prognosis of Extensive-Stage Small-Cell Lung Cancer

**DOI:** 10.1155/2020/8811487

**Published:** 2020-11-19

**Authors:** Dongfang Chen, Jianlin Xu, Rong Qiao, Yizhuo Zhao, Tianqing Chu, Baohui Han, Runbo Zhong

**Affiliations:** Department of Pulmonary Medicine, Shanghai Chest Hospital, Shanghai Jiaotong University, Shanghai, China

## Abstract

Some studies have revealed that specific genetic mutations could be associated with chemotherapy response or even survival in small-cell lung cancer (SCLC). Our retrospective study aimed to identify the correlation between genetic mutations and progression-free survival (PFS) in extensive-stage SCLC after first-line chemotherapy. A total of 75 patients with extensive-stage SCLC confirmed by histopathology from February 2018 to February 2019 were retrospectively analyzed. The biopsy specimens of all patients were analyzed by Next-Generation Sequencing (NGS). All patients received first-line chemotherapy and follow-up at Shanghai Chest Hospital. Eleven genes were mutated in, at least, 10% of the 75 patients, including *TP53* (96%), *RB1* (77%), *SMAD4* (32%), *NOTCH1* (21%), *PTEN* (16%), *FGFR1* (16%), *KDR* (15%), *PIK3CA* (15%), *ROS1* (15%), *BRCA2* (13%), and *ERBB4* (10%). The median number of mutated genes among all patients was 5. Patients with more than 5 mutated genes (PFS = 6.7 months, *P*=0.004), mutant *TP53* (PFS = 5.0 months, *P*=0.011), and mutant *BRCA2* (PFS = 6.7 months, *P*=0.046) had better PFS after first-line chemotherapy than other patients. Multivariate Cox regression analysis showed that patients who achieved a PR (HR 3.729, 95% CI 2.038–6.822), had more than 5 mutated genes (HR 1.929, 95% CI 1.096–3.396), had *BRCA2* mutations (HR 4.581, 95% CI 1.721–12.195), and had no liver metastasis (HR 0.415, 95% CI 0.181–0.951) showed improvements in PFS after first-line chemotherapy. In conclusion, the number of mutated genes and *BRCA2* mutation status in extensive-stage SCLC were significantly related to PFS after first-line chemotherapy.

## 1. Introduction

Small-cell lung cancer (SCLC) is a malignant neuroendocrine tumor with an epithelial source and accounts for approximately 15% to 17% of all diagnosed lung cancers [1]. The unique biological characteristics of SCLC include a close association with smoking, rapid proliferation, and early hematogenous metastasis [2]. Thus, 80% of patients are at an extensive stage when first diagnosed. The liver, bone, kidney, and brain are several common distant metastasis sites. For these extensive-stage patients, the current first-line standard chemotherapy is etoposide plus carboplatin or cisplatin [3]. The median survival of patients with advanced SCLC with chemotherapy is between 7 and 10 months; the one-year overall survival (OS) rate is approximately 20% to 40% [1]. Except for the ALTER 1202 and IMpower133 trials, there have been a few advances in targeted therapy and immunotherapy for SCLC over the past 30 years. The novel multitarget tyrosine kinase inhibitor (TKI) anlotinb which targets vascular endothelial growth factor receptor type 2 and 3 (VEGF 2, 3), platelet-derived growth factor *β* (PDGFR*β*), fibroblast growth factor receptor (FGFR), and stem cell-factor receptor (c-Kit) has been approved as a third-line or beyond therapy for SCLC by the National Medical Products Administration (NMPA) based on the ALTER 1202 trial in 2019 [4]. In the IMpower133 trial, OS following the atezolizumab-based combination was only two months longer than that following chemotherapy alone [5]. However, chemotherapy is still an important component of standard first-line therapy for extensive-stage SCLC.

The emergence of NGS has revolutionized the detection of genetic mutations. The advantages of NGS are that it allows high throughput analysis, has good sensitivity, and provides abundant information [6]. With the application of NGS technology, many studies have revealed that genetic mutation rates are extremely high in SCLC [7–9]. Several driver genes or signaling pathways may be simultaneously activated during SCLC development, which promotes tumor progression through different mechanisms. *TP53* and *RB1* mutations exist in almost all SCLCs [10]. Other common mutated genes include *MYC* family members, *FGFR1*, *SOX2*, *PIK3CA*, *PTEN*, and *NOTCH* family members [11].

Most patients with extensive-stage SCLC have relatively good responses to first-line chemotherapy, but they will eventually experience chemotherapy resistance and relapse. According to NCCN guidelines, SCLC patients who relapse beyond three months after first-line chemotherapy are categorized as chemosensitive, whereas those who relapse within three months of initial chemotherapy are considered chemoresistant. Only a few studies have revealed that certain mutated genes are associated with chemotherapy sensitivity. Chinese scholars identified *NDRG4* as a new tumor suppressor gene that plays a tumor suppressive role in SCLC. SCLC patients with *NDRG4* mutations are more sensitive to chemotherapy and have a longer survival time than those without such mutations, which suggests that the NDRG4 protein could be used as a biomarker to predict the clinical prognosis of SCLC. Another study focused on cisplatin-resistant target genes and genes associated with poor prognosis in SCLC, and the results indicated that *DNAH10* mutations were significantly associated with cisplatin resistance, poor OS, and worse PFS in SCLC [12]. Therefore, *DNAH10* mutations may have potential value in predicting cisplatin resistance and poor survival in SCLC.

The abovementioned studies suggest that specific genetic mutations may be associated with chemotherapy response or survival in SCLC. Since gene-related research on SCLC is limited, the relationship between genetic mutations and survival in SCLC is worth further study. This study may be helpful for identifying SCLC patients who could benefit from chemotherapy, which is of great practical significance. Thus, the main purpose of our retrospective study is as follows: first, to investigate the mutation status of extensive-stage SCLC patients, including nonsmoking and female patients, and second, to identify the correlation between genetic mutations and PFS in extensive-stage SCLC patients after first-line chemotherapy.

## 2. Materials and Methods

### 2.1. Patients

We identified and reviewed the clinical data of patients who were diagnosed with extensive-stage SCLC at Shanghai Chest Hospital from February 2018 to February 2019. The study protocol was approved by the Ethics Committee of Shanghai Chest Hospital (KS1934). The key inclusion criteria were as follows: (1) patients who were diagnosed with SCLC histopathologically and at an extensive stage clinically; (2) patients who had, at least, one measurable tumor lesion; (3) patients whose biopsy specimens were analyzed by NGS; (4) patients who were between 18 and 70 years of age; (5) patients whose Eastern Cooperative Oncology Group performance status was 0 or 1; and (6) patients who received etoposide plus carboplatin or cisplatin as first-line chemotherapy until disease progression or intolerance to chemotherapy. The key exclusion criteria were as follows: (1) patients who were diagnosed with limited-stage SCLC; (2) patients who had severe and/or uncontrolled diseases before first-line chemotherapy; and (3) patients who suffered from other malignant tumors simultaneously.

### 2.2. Clinical Assessments

Patients were given etoposide (100 mg/m^2^) plus carboplatin (AUC = 5) or cisplatin (75 mg/m^2^) at every course. Clinical follow-up exams included a physical examination and laboratory tests, which were performed at every course. Efficacy was evaluated every two courses by computed tomography according to Response Evaluation Criteria in Solid Tumors (RECIST v1.1) and included complete response (CR), partial response (PR), stable disease (SD), and progressive disease (PD). If the first-line treatment failed, a second-line treatment was given. All patients were regularly followed up after receiving first-line chemotherapy at Shanghai Chest Hospital. The follow-up ended on April 10, 2019.

### 2.3. NGS

The llumina TruSeq Amplicon Cancer Panel kit (Burning Rock Company, China) and the MiSeq instrument were used for sequencing. The sequencing targeted the mutation hotspots of 68 genes with high mutation rates in lung cancer (Table 1). The main steps were as follows: (1) an NGS library was prepared by fragmenting a gDNA sample and ligating specialized adapters to both fragment ends; (2) the library was loaded into a flow cell and the fragments hybridized to the flow cell surface, and each bound fragment was amplified into a clonal cluster through bridge amplification; (3) sequencing reagents, including fluorescently labeled nucleotides, were added, the first base was incorporated, the flow cell was imaged, the emission from each cluster was recorded, the emission wavelength and intensity were used to identify the base, and this cycle was repeated “*n*” times to create a read length of “*n*” bases; and (4) data analysis.

### 2.4. Statistical Analysis

Progression-free survival (PFS) was measured from the date of initiation of first-line chemotherapy to the date of disease progression or the last follow-up visit. SPSS22.0 statistical software (IBM, Armonk, NY, USA) was used for data processing. Only genes that were mutated in, at least, 10% of the enrolled patients were considered for statistical analysis. The Mann–Whitney *U* test was used to identify differences in the number of mutated genes between groups. The Kaplan–Meier method and log-rank test were used to identify the correlation between genetic mutations and PFS. Multivariable Cox regression was used to identify significant factors related to PFS. All tests were two sided, and *P* values <0.05 were considered statistically significant.

## 3. Results

### 3.1. Patient Characteristics

The study sample consisted of 75 extensive-stage SCLC patients, 65 (87%) of which were smokers and 10 (13%) of which were never-smokers. After first-line chemotherapy, 44 (59%) patients achieved a PR, 23 (31%) patients had SD, and the remaining 8 (10%) patients had PD. The proportions of patients with bone metastasis, brain metastasis, and liver metastasis were 24%, 13%, and 15%, respectively. Demographic data for all patients are shown in Table 2.

### 3.2. Genetic Mutations

Eleven genes were mutated in, at least, 10% of the 75 patients. The top two common mutated genes were *TP53* (96%) and *RB1* (77%). *SMAD4* and *NOTCH1* were mutated in 32% and 21% of the 75 patients, respectively. The frequencies of mutated genes related to the PIK3-AKT-mTOR signaling pathway (*PTEN*, *FGFR1*, and *PIK3CA*) were similar. Other mutated genes are shown in Table 3.

The most frequent mutated genes in nonsmoking patients were *TP53* (100%), *RB1* (73%), *SMAD4* (35%), *KDR* (23%), *NOTCH1* (20%), and *PTEN* (20%). The most frequently mutated genes in female patients were *TP53* (100%), *RB1* (78%), *SMAD4* (56%), *NOTCH1* (44%), *KDR* (44%), *NTRK1* (33%), and *RET* (22%).

The number of mutated genes among all patients ranged from 2 to 15, and the median was 5. There were no significant differences between the gender, age, smoking status, and anatomy type (central type/peripheral type) subgroups (Table 4).

### 3.3. PFS

**Figure 1:**
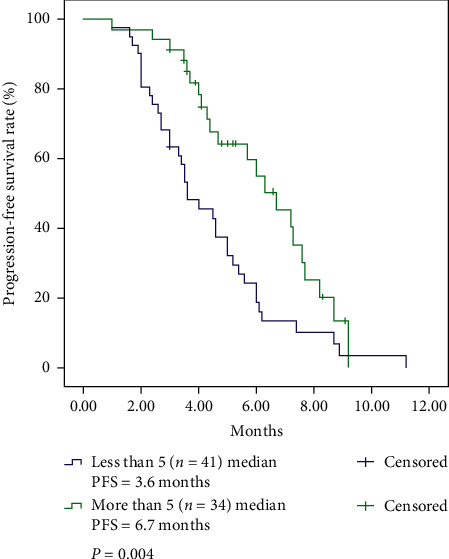
Kaplan–Meier curves of progression-free survival based on the number of mutated genes.

The median PFS of the 75 patients was 4.7 months (95% CI 3.8–5.5). The median PFSs of male and female patients were 4.6 months (95% CI 3.6–5.6) and 6.0 months (95% CI 4.0–8.0), respectively (*P*=0.777). The median PFSs of never-smokers and smokers were 5.2 months (95% CI 3.9–6.5) and 4.6 months (95% CI 3.1–6.1), respectively (*P*=0.285). The relationship between genetic mutations and PFS was investigated. Surprisingly, patients with more than 5 mutated genes had a better PFS than patients with less than 5 mutated genes (6.7 versus 3.6 months, *P*=0.004) (Figure 1). Among the mutated genes listed in Table 2, patients with mutant *TP53* had a better PFS than those with wild-type *TP53* (5.0 versus 3.4 months, *P*=0.011). Similarly, patients with *BRCA2* mutations had a better PFS than patients with wild-type *BRCA2* (6.7 versus 4.5 months, *P*=0.046) (Table 5).

Given the high frequencies of *TP53* and *RB1* mutations, the correlation between mutation abundance and PFS was further analyzed. Patients with mutant *TP53* were divided into two groups according to the mutation abundance (≤80% and >80%, median: 80%), and there was no significant difference in PFS between the two groups (*P*=0.803). Additionally, there was no significant difference in PFS related to the abundance of mutant *RB1* (≤77% and >77%, median: 77%; *P*=0.372).

Multivariate Cox regression analysis showed that the response to first-line chemotherapy, the number of mutated genes, *BRCA2* mutation status, and liver metastasis had significant effects on PFS after first-line chemotherapy (Table 6). Patients who achieved a PR (HR 3.729, 95% CI 2.038–6.822), had more than 5 mutated genes (HR 1.929, 95% CI 1.096–3.396), had *BRCA2* mutations (HR 4.581, 95% CI 1.721–12.195), and had no liver metastasis (HR 0.415, 95% CI 0.181–0.951) showed improvements in PFS.

## 4. Discussion

There are only few studies that have investigated the association of genetic mutations with clinical prognosis in extensive-stage SCLC. Therefore, we collected data from 75 extensive-stage SCLC patients for further study. Our study revealed the mutation status of extensive-stage SCLC patients, especially nonsmoking and female patients. In addition, we also found that the genetic mutations of extensive-stage SCLC were related to PFS after first-line chemotherapy.

In our study, the genes that mutated in more than 20% of the total patients were *TP53, RB1*, *SMAD4*, and *NOTCH1*. In previous reports, *TP53* and *RB1* mutations were shown to affect up to 90% and up to 65% of SCLC patients, respectively [13]. The importance of these two mutated genes in SCLC tumorigenesis has been highlighted by numerous functional studies [14]. The frequency of *SMAD4* mutation was surprisingly high in our study. *SMAD4* mediates the signaling of transforming growth factor beta and bone morphogenic protein ligands and is a well-defined tumor suppressor in pancreatic and colon cancer [15]. *SMAD4* mutations are associated with lymph node metastases, increased angiogenesis, and more aggressive cellular behavior in vitro [15]. *NOTCH* signaling is critical for the regulation of neuroendocrine differentiation. A previous study identified the presence of mutations in *NOTCH* family members in a quarter of analyzed SCLC samples [16], which was confirmed in our study.

SCLC is strongly correlated with a history of smoking and mainly occurs in males, but a small portion of patients are nonsmoking or female. It is meaningful to explore the mutation status of this special population. We found that *TP53*, *RB1*, *SMAD4*, *KDR*, and *NOTCH1* were mutated frequently in both nonsmoking and female patients. This could be explained by the fact that most of the female patients were never-smokers. In addition, nonsmoking patients had a high prevalence of *PTEN* mutations, while female patients had a high prevalence of *NTRK1* and *RET* mutations. Cardona et al. reported that, among 10 never-/ever-smokers, the most frequent genetic mutations detected by NGS were *TP53* (80%), *RB1* (40%), *CYLD* (30%), *EGFR* (30%), *MET* (20%), *SMAD4* (20%), and *BRIP1* (20%) [17]. In a study by Sun et al., among 28 genetically evaluable never-smokers, the most common mutations included TP53 (93%), RB1 (25%), PTEN (18%), *EGFR* (14%), MET (14%), and SMAD4 (11%) [18]. Our data and previous findings are not entirely consistent with each other, since differences exist in terms of sample size, sequencing panel, and tumor stage.

Smoking status and gender were not shown to be related to PFS in our study. In an analysis of 20 SCLC patients, an improvement in survival in terms of PFS in response to first-line treatment according to smoking status was not observed, but never-smokers achieved an improvement in OS compared to smokers [17]. In another study, in 394 extensive-stage SCLC patients who received first-line chemotherapy, both PFS and OS were correlated with smoking history [19]. Overall, according to previous studies, smoking seems to be a negative factor for survival outcomes, especially shorter OS. While gender-related differences in PFS were not seen according to our data, gender may play an important role in the prognosis of SCLC. Dowlati et al. found that female sex was a positive factor for response to chemotherapy in extensive-stage SCLC patients [20]. A pooled analysis of randomized SCLC chemotherapy trials showed that female patients survived modestly longer than male patients [21]. Another study indicated that female sex is useful as a predictor for better long-term survival [22].

Tumor mutational burden (TMB), a quantification of tumoral mutations, has been associated with the response to immunotherapy. In the CheckMate 032 trial, 401 patients received treatment with nivolumab or a combination of nivolumab and ipilimumab [23]. Among patients treated with combination therapy, a high TMB was related to better ORR and OS. In the CheckMate 227 trial, first-line nivolumab plus ipilimumab significantly prolonged PFS versus chemotherapy in advanced NSCLC patients with a high TMB (≥10 mutations/megabase) [24]. Thus, TMB may be a prognostic factor for lung cancer immunotherapy. In contrast to previous studies, blood-based TMB showed no value in predicting benefit with atezolizumab in the IMpower133 trial, and the possible explanation was that the combination of platinum and etoposide was active and myelosuppressive [5]. The KEYNOTE 604 trial showed that pembrolizumab combined with standard first-line EP significantly improved PFS in extensive-stage SCLC patients, but TMB was not further analyzed [25]. Notably, our study showed that an improvement in PFS was observed in patients who had more than 5 mutated genes. We may further make an assessment of TMB to verify this conclusion. Additionally, more basic medical research is needed to reveal the specific mechanism of our finding.

In our retrospective study, among 11 common mutated genes, mutant *TP53* and *BRCA2* were associated with better PFS. Using multivariate analysis, only *BRCA2* was significant in predicting PFS. Dowlati et al. reported that patients with *TP53* mutations had similar PFS and OS as patients with wild-type *TP53* [20]. However, only 3 (4%) patients had wild-type *TP53*, and this result should be confirmed in a larger sample. Dowlati et al. also reported that patients with mutant *RB1* had both better OS and PFS than patients with wild-type *RB1*, but this was not validated in the multivariate analysis. *BRCA2* helps repair damaged DNA and plays a crucial role in ensuring the stability of genome. Patients with *BRCA2* mutations may develop genetic alterations leading to cancer. Specific inherited mutations in *BRCA2* may notably increase the risk of female breast and ovarian cancers, but they have also been associated with increased risks of several additional types of cancer. There is little research indicating a relationship between mutant *BRCA2* and SCLC, but our finding suggests that there might be interactions between mutant *BRCA2* and SCLC.

Moreover, other similar studies are worth referencing. A group of researchers found that *CREBBP/EP300, TP73*, or *NOTCH* mutations had no influence on the survival of SCLC patients treated with surgery and chemotherapy [14]. A chromogenic in situ hybridization study showed that *MYC* amplification was associated with poor survival in SCLC and might be an independent prognostic factor for SCLC [26].

Our study has some limitations. First, the sample size is small. Second, the OS data are not shown because of the high loss ratio of follow-up.

## 5. Conclusions

In our retrospective study, the number of mutated genes and *BRCA2* mutation status in extensive-stage SCLC were significantly related to PFS after first-line chemotherapy.

## Figures and Tables

**Table 1:** The 68 genes detected by NGS.

ALK	BRAF	EGFR	ERBB2	KRAS	MET	RET	ROS1
AKT1	APC	ARAF	ATM	AXL	BCL2L11	BRCA1	BRCA2
CCND1	CD74	CDK4	CDK6	CDKN2A	CTNNB1	DDR2	ERBB3
ERBB4	ESR1	FGF19	FGF3	FGF4	FGFR1	FGFR2	FGFR3
FLT3	HRAS	IDH1	IDH2	IGF1R	JAK1	JAK2	KDR
KIT	MAP2K1	MTOR	MYC	NF1	NOTCH1	NRAS	NRG1
NTRK1	NTRK2	NTRK3	PDGFRA	PIK3CA	PTCH1	PTEN	RAF1
RB1	SMAD4	SMO	STK11	TOP2A	TP53	TSC1	TSC2
AR	CYP2D6	DPYD	UGT1A1				

**Table 2:** Characteristics of the 75 extensive-stage SCLC patients.

Characteristic	*N* (%)
Gender	
Male/female	66 (88)/9 (12)
Age	
<65/≥65	39 (52)/36 (48)
Smoking status	
Never-smoker/smoker	10 (13)/65 (87)
Anatomy type	
Central type/peripheral type	55 (74)/20 (26)
Response to first-line chemotherapy	
PR/SD/PD	44 (59)/23 (31)/8 (10)
Metastatic sites	
Bone	18 (24)
Brain	10 (13)
Liver	11 (15)
Lung (contralateral)	28 (37)
Pleura	20 (27)
Pericardium	3 (4)
Kidney	3 (4)

SCLC, small-cell lung cancer; PR, partial response; SD, stable disease; PD, progressive disease.

**Table 3:** Frequency of genes mutated in, at least, 10% of the 75 patients.

Gene	Mutant *N* (%)
*TP53*	72 (96)
*RB1*	58 (77)
*SMAD4*	24 (32)
*NOTCH1*	16 (21)
*PTEN*	12 (16)
*FGFR1*	12 (16)
*KDR*	11 (15)
*PIK3CA*	11 (15)
*ROS1*	11 (15)
*BRCA2*	10 (13)
*ERBB4*	8 (10)

**Table 4:** Number of mutated genes in the different subgroups.

Subgroup	*N* (median, range)	*P* value
Gender		
Male/female	5 (2–15)/5 (3–12)	0.216
Age		
<65/≥65	5 (2–13)/6 (2–15)	0.209
Smoking status		
Never-smoker/smoker	5 (3–12)/5 (2–15)	0.857
Anatomy type		
Central type/peripheral type	5 (2–15)/5 (3–13)	0.884

**Table 5:** Effect of mutation status on PFS after first-line chemotherapy.

Gene	Median PFS in months (95% CI)	*P* value
*TP53*		0.011
WT	3.4 (1.2–5.6)	
Mutant	5.0 (4.1–5.9)	
*RB1*		0.576
WT	6.0 (2.9–9.1)	
Mutant	4.7 (4.0–5.4)	
*SMAD4*		0.077
WT	4.1 (3.1–5.1)	
Mutant	6.3 (4.9–7.7)	
*NOTCH1*		0.191
WT	4.6 (3.4–5.8)	
Mutant	6.0 (3.1–8.9)	
*PTEN*		0.666
WT	4.7 (3.8–5.6)	
Mutant	5.0 (3.0–7.0)	
*FGFR1*		0.472
WT	4.7 (4.0–5.4)	
Mutant	5.7 (3.2–8.2)	
*KDR*		0.522
WT	4.6 (3.7–5.5)	
Mutant	5.4 (3.3–7.5)	
*PIK3CA*		0.613
WT	4.6 (3.7–5.5)	
Mutant	6.0 (3.0–9.0)	
*ROS1*		0.608
WT	4.7 (3.8–5.6)	
Mutant	5.7 (2.8–8.6)	
*BRCA2*		0.046
WT	4.5 (3.6–5.4)	
Mutant	6.7 (5.0–8.4)	
*ERBB4*		0.660
WT	4.6 (3.9–5.3)	
Mutant	6.0 (4.5–7.5)	

PFS, progression-free survival; WT, wild type; CI, confidence interval.

**Table 6:** Multivariate Cox regression analysis of PFS after first-line chemotherapy.

Factor	*P* value	HR (95% CI)
Age	0.201	
Bone metastasis	0.353	
*TP53*	0.068	
*SMAD4*	0.412	
*NOTCH1*	0.073	
Response to first-line chemotherapy	<0.0001	3.729 (2.038–6.822)
Number of mutated genes	0.023	1.929 (1.096–3.396)
*BRCA2*	0.002	4.581 (1.721–12.195)
Liver metastasis	0.038	0.415 (0.181–0.951)

PFS, progression-free survival; HR, hazard ratio;CI, confidence interval.

## Data Availability

The data used to support the findings of this study are available from the first author and the corresponding author upon request.
